# Lengthening over nails using the double plate system ONAS-DPS

**DOI:** 10.1051/sicotj/2015040

**Published:** 2016-02-02

**Authors:** Vane Antolič

**Affiliations:** 1 Department of Orthopaedic Surgery, University Medical Centre and Faculty of Medicine Ljubljana Slovenia

**Keywords:** Lengthening over nail, External fixation, Intramedullary nail, Angle stability plate

## Abstract

Stable insertion of large Schanz screws behind an intramedullary (IM) nail when lengthening over nails (LON) may be difficult due to the limited bone stock. Additionally, the highly probable contact between the screws and IM nail (which is difficult to avoid) increases the likelihood of infection spreading from the skin via Schanz screws directly to the IM nail. A new device for LON has been developed. Instead of inserting Schanz screws from the external fixator beside the IM nail (as in standard LON), a system of two overlaying plates was constructed. Schanz screws can be fixed to the plates without entering the bone. The plates are fixed to the bone using four angle stability screws. The holes in the plates offer stabile fixation for a chosen angle under which the screw is positioned through the cortical bone. Using the new system there is no need to place Schanz screws behind the IM nail. Instead, Schanz screws pass to the plate and not through the bone. The new system for elongation over IM nail is called “Over Nail Angle Stability-Double Plate System” (ONAS-DPS) [Antolič V (2013) Modular side device with an intramedullary nail for guiding a bone during its lengthening. World Intellectual Property Organization. International Publication number: WO 2013/176632 A1].

## Introduction

Elongation of long bones can be achieved by using external fixation, intramedullary nails (IM) or combination of both, i.e. lengthening over nail (LON). In external fixation, pin tract infection is a problem due to many factors, including the time pins which are required to stay in place in order to achieve adequate bone regenerate. Other problems are malalignment and poor control of elongation, delayed bone formation, refracture, adjacent joint problems, etc. [1–3]. Overall complication rate may be as high as 75% [2] or even 100% [4] and as much as 1.2 per bone [2].

LON was a step forward in the long bone elongation philosophy [5–8]. The advantages of LON include a decrease in the duration of external fixation, better alignment, protection against refracture and earlier rehabilitation [6–9]. Although LON can reduce the duration of external fixation and malalignment, caution is required to prevent major complications, i.e. infection [10–12]. In LON, screws (at least two) must be placed beside (typically behind) the IM nail with no contact between the screws and the IM nail (if possible) in order to prevent potential infection from the skin via screws directly to the IM nail. The problem is that the bone stock is always limited around the IM nail and it is quite difficult to place the screws properly. The necessity for strong and stable fixation needed for a successful LON, places an additional burden on the surgeon. Besides, it is expected that each screw is placed in the right position on the first attempt: further attempts decrease the likelihood of achieving the correct position and stability of the screw. In LON, the infection rate was reported as 5/9 children [13] and the infection rate was similar in the study by Kim et al. in 2011 [14].

Automated IM nails completely avoid external fixation and are supposed to reduce the infection rate. However, they show some other limitations, i.e. “runaway nail” [15], mechanical failures [16, 17], insufficient bone regeneration and high overall complication rates [18]. Femoral lengthening with LON has fewer complications than IM skeletal kinetic distraction [9]. There are many arguments against the use of automated nails [18]. In comparison to automated nail techniques, LON offers more control during lengthening and less technology-related failures, i.e. “runaway and blockage”. Besides, in the LON group the complication rate was 5% whereas in the automated IM nail group the complication rate was 50% [9]. On the other hand, complications of the automated IM nails might be related to the nail technology itself. Some more promising results have been reported with the new IM nail technology, but with a minimum follow-up of only three weeks [19].

## Surgical technique

A new device: ONAS-DPS (Over Nail Angle Stability-Double Plate System).

**Figure 1.**
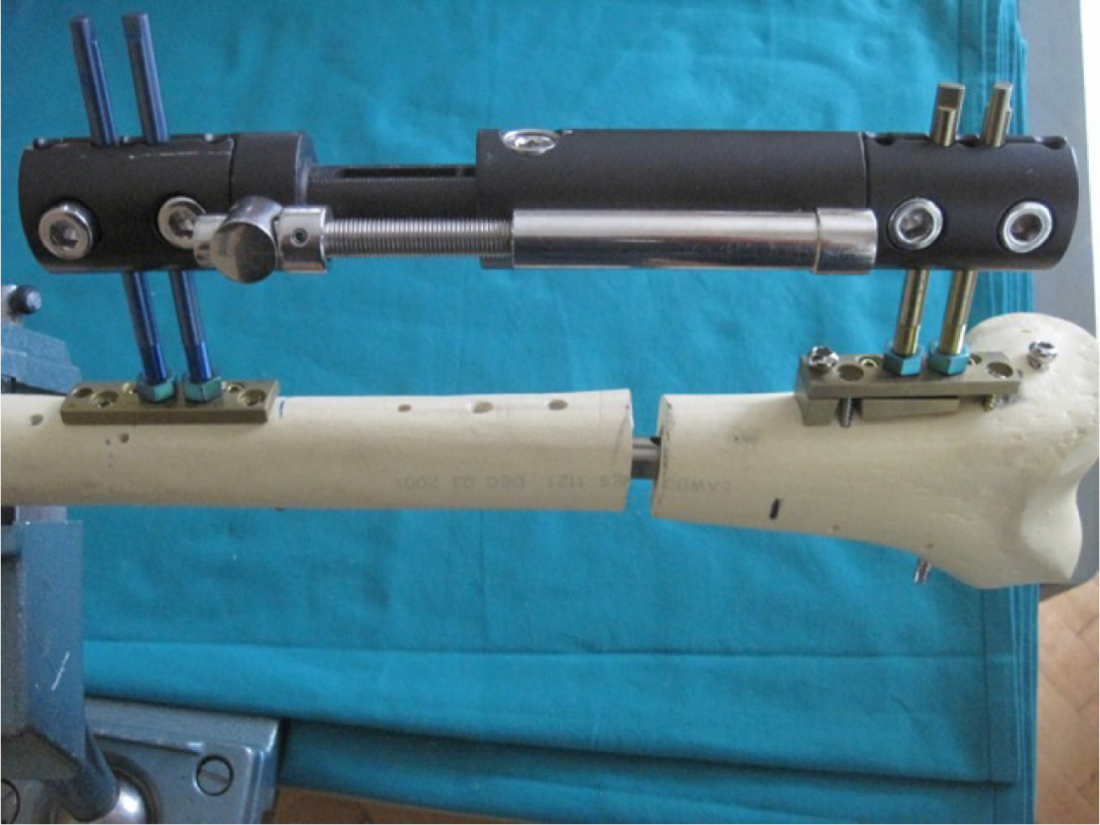
ONAS-DPS: a retrograde femoral IM nail and unilateral frame are shown on the bone model. Two Schanz screws are fixed proximally and distally (Figures 2 and 3) into the plate. Note that the screws are not entering the underlying cortical bone and in this way the contact with IM nail is avoided. A wedge subplate is used distally. In the case of anterograde IM femoral nail (not shown) the “wedge plate” is used proximally. The most distal screw goes through the IM nail in order to provide adequate stability of the whole system.

**Figure 2.**
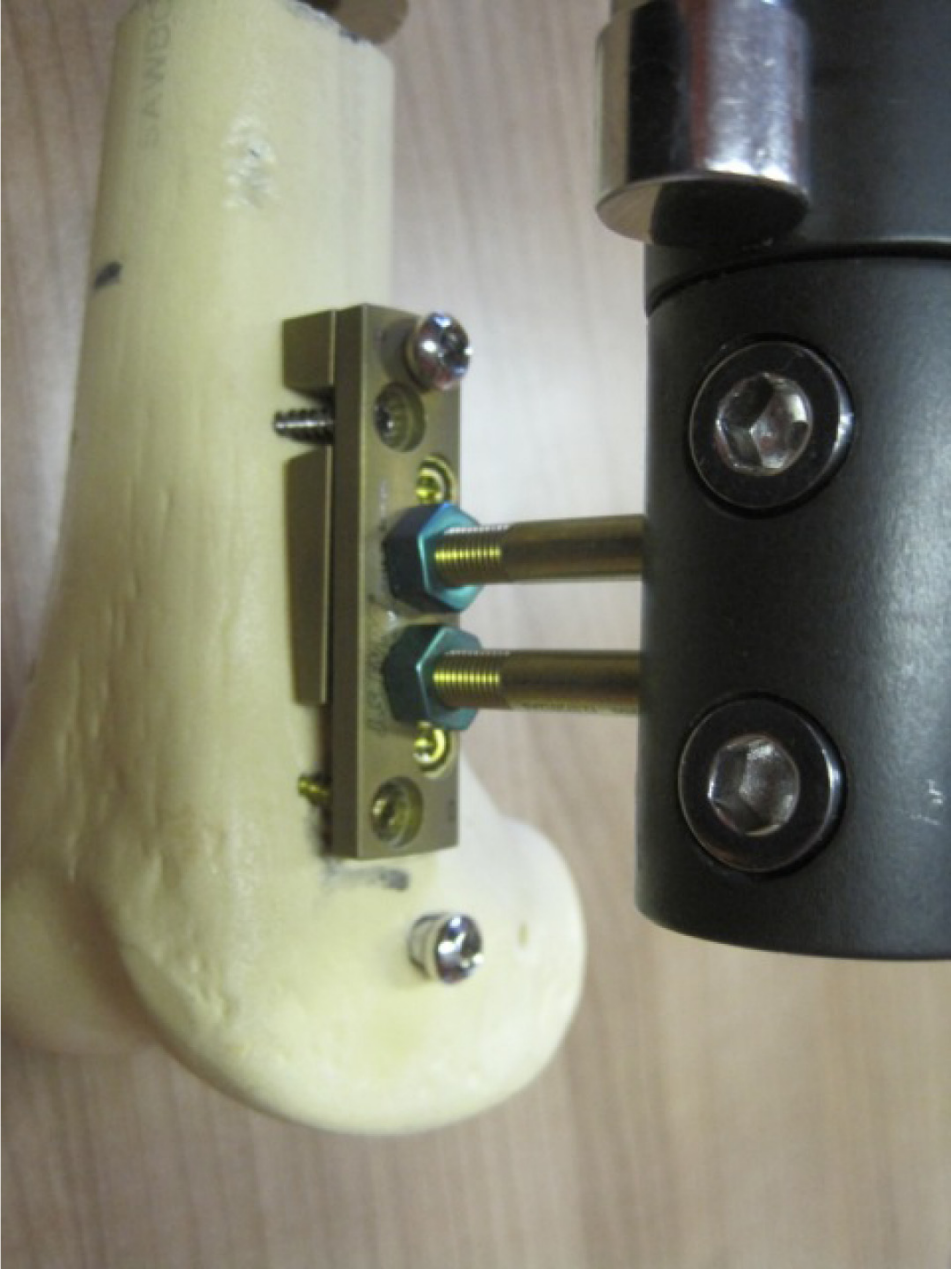
ONAS-DPS. Two plates are needed distally (Figure 1) since the femoral metaphysis is curved: the lower plate (subplate) is a specially designed “wedge plate” which makes the unilateral external fixator parallel to the bone. If ONAS-DPS is used with anterograde IM nail the wedge plate must be used proximally (not shown). Wedge plates with four different angles (thicknesses) are available with ONAS-DPS, each having an equivalent probe. The upper plate provides fixation to the bone with four angle stability screws. Each screw hole in the plate offers the possibility of placing the screw at any chosen angle from 90° to 65° with respect to the sagittal axis of the bone. Please note that the IM nail is in the medullary canal and that the plates are fixed with screws going anteriorly and posteriorly with respect to the IM nail. Proximally, a separate screw passes through both plates and cortical bone and goes through the IM nail and finally anchors into the opposite cortical bone. The two-plate system of the ONAS-DPS enables stable fixation of Schanz screws and also stable fixation of both plates to the bone, in this way avoiding direct contact between Schanz screws and IM nail, which is responsible for the high incidence of infection in standard LON.

**Figure 3.**
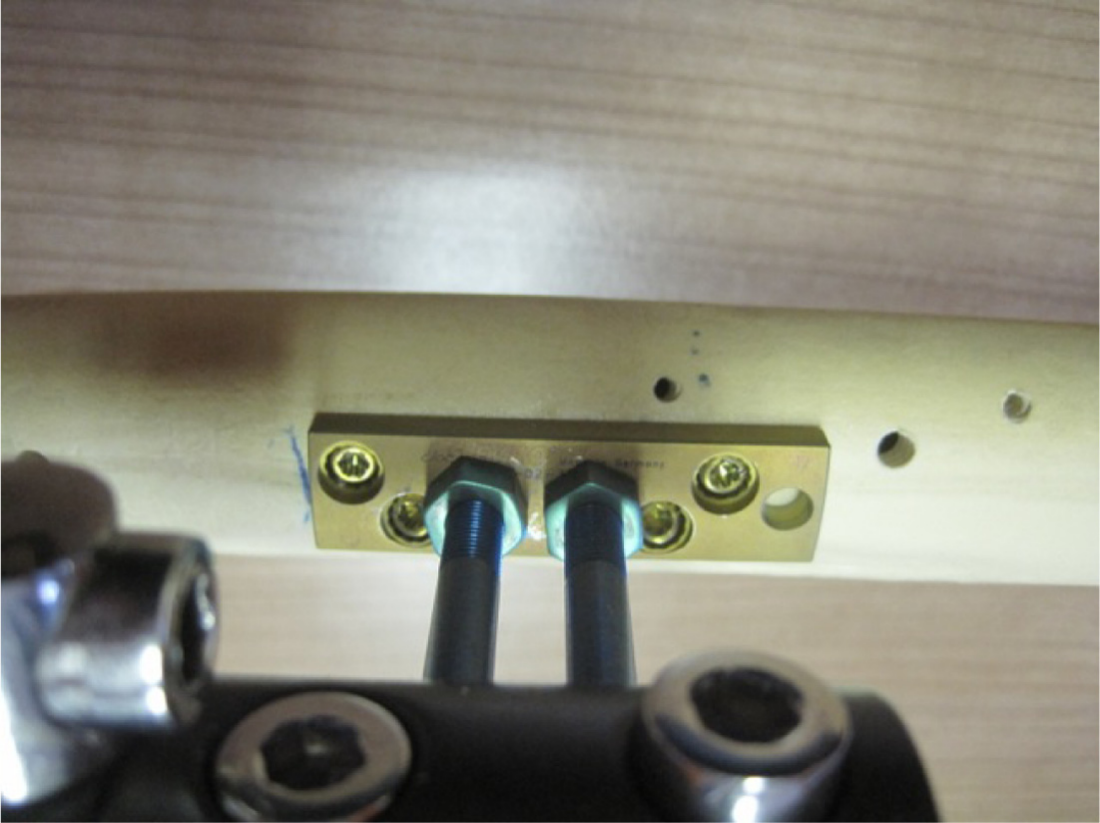
Proximal fixation of Schanz screws in the case of retrograde technique (Figure 1). On the diaphysis one plate of the ONAS-DPS can be used only (distally a wedge subplate must be used in order to compensate for the femoral shape – Figures 1 and 2). Further, no screw should go through the IM nail proximally (in contact with the distal fixation – Figures 1 and 2) as this would prevent elongation. Four angle stability screws and asymmetrically drilled corresponding screw holes are the same as shown in Figure 2.

**Figure 4.**
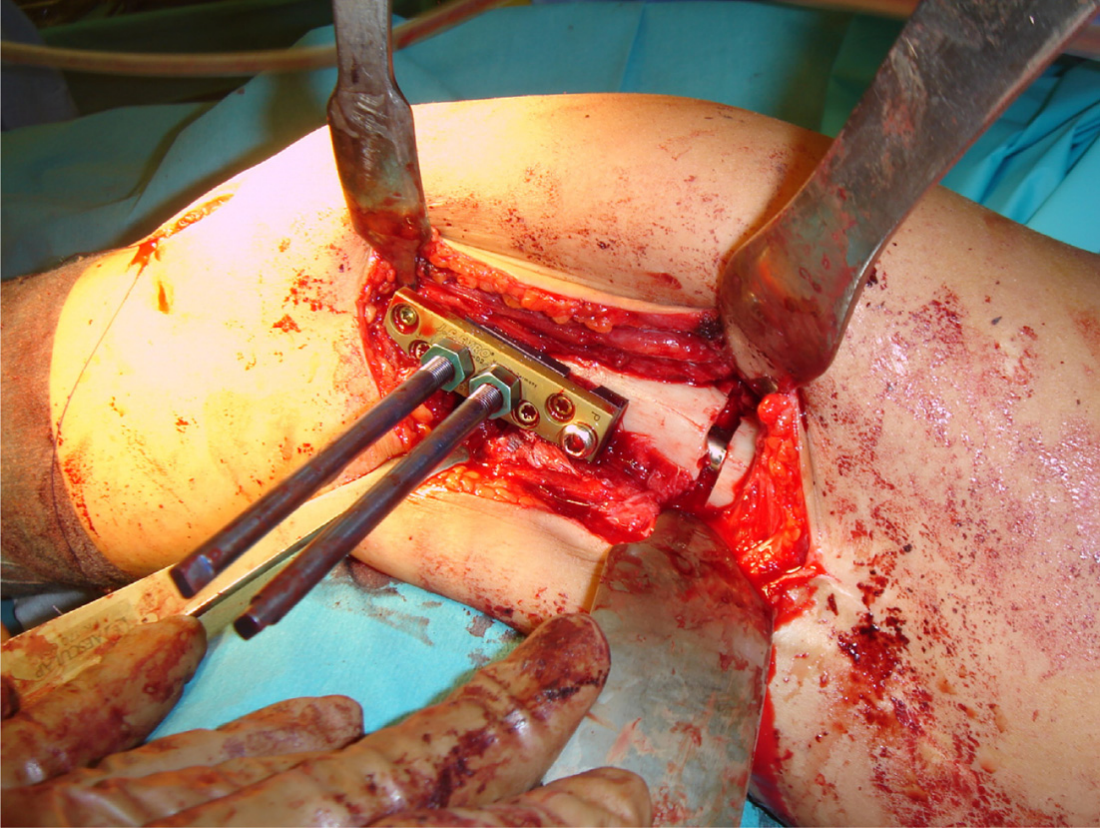
Intraoperative view of ONAS-DPS implantation in a 28-year-old female patient with congenital femoral shortening and deformity (osteotomy, intramedullary nail, plate with two Schanz screws). Note that there is no direct contact between Schanz screws and the bone or the IM nail.

**Figure 5.**
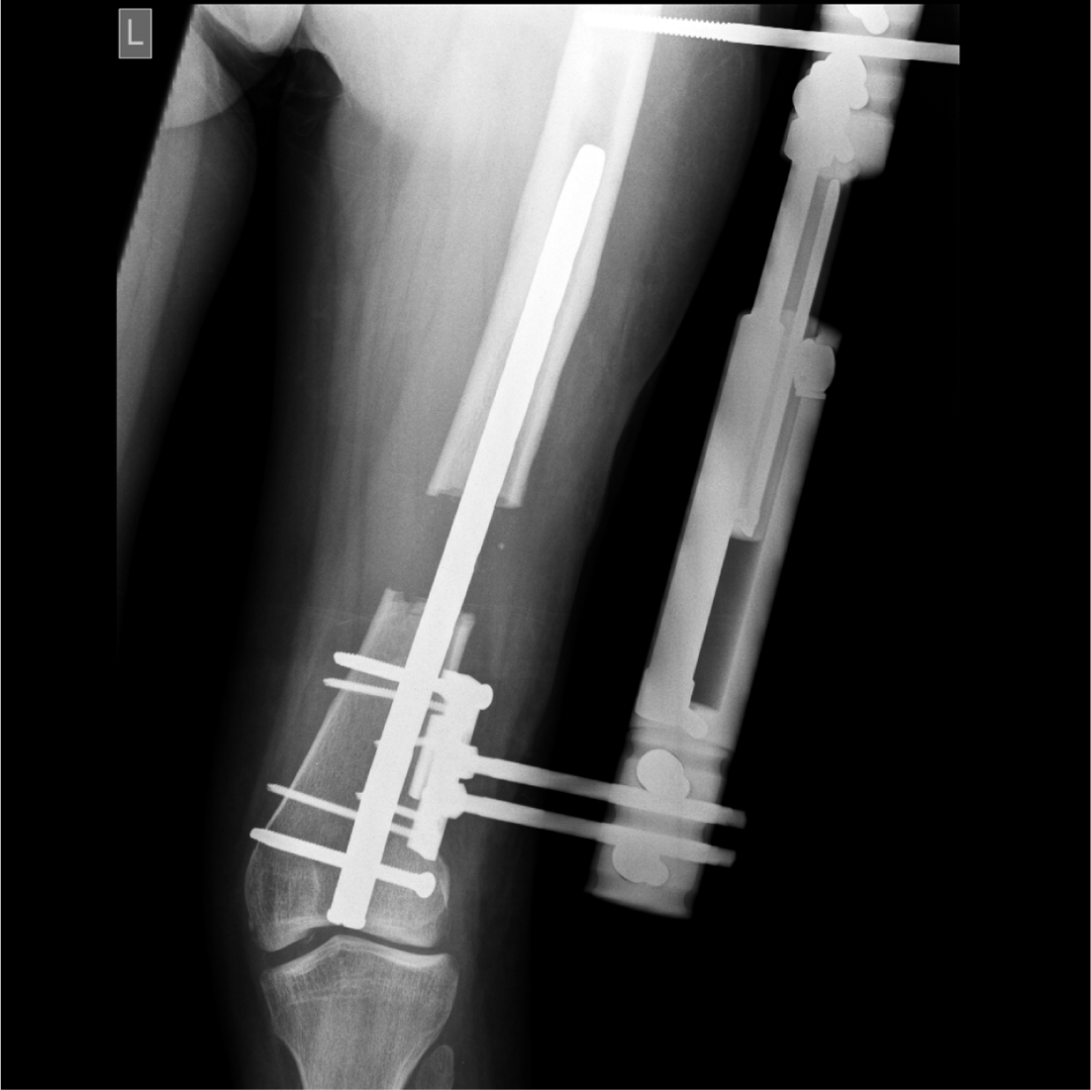
Radiograph of the distal femur in the patient from Figure 4 after the acute phase of lengthening has been completed.

The “Over Nail Angle Stability-Double Plate System” (ONAS-DPS) has been developed for elongation over IM nail (Figures 1–3) with the intention of preserving the LON idea and all of its advantages. On the other hand, the aim of the new device is to reduce the deep infection rate, which proved to be the major problem of LON. The system is patented, has CE certificate and is produced in Germany, European Union (info: vane.antolic@guest.arnes.si, www.antolic.si). With the ONAS-DPS there is no need to use the Schanz screws beside the IM nail, which might be a technical problem during surgery due to the limited bone stock (Figures 2 and 3). ONAS-DPS enables the fixation of two specially designed Schanz screws directly to the plate (Figures 2 and 3). The screws go as far as the “angle stability plate system” (and not through the bone), in this way avoiding the necessity of drilling Schanz screws through both cortices. This way the contact between the Schanz screws and the IM nail is also avoided (Figures 4 and 5). Adequate stability is easily achieved by screwing the Schanz screws into the plate overlaying the bone. Although the rate of pin track infection itself cannot be reduced, the fact that Schanz screws do not touch the intramedullary nail directly may reduce the risk of deep bone infection. As in LON, after achieving the desired degree of elongation (Figure 5) the ONAS-DPS (with possible bacterial contamination) and the external fixator are removed and the intramedullary nail is locked.

Standard surgical technique for retrograde approach through the knee joint is used: entering through mini incision and drilling in the notch under C-arm control (Figure 5). A standard Orthofix unilateral fixator provides external fixation and the callotasis technique is used for elongation. Lengthening is started on the 7th postoperative day at a rate of one quarter of a millimeter four time per day. The unilateral fixator is removed when lengthening ceases. An identical technique could also be applicable for use in the tibia.

## Mechanical testing

A push-out test was performed with 2.7 mm locking screws in order to test the mechanical strength required to extract perpendicularly applied screw from the flat polyethylene surface. The results are shown in Table 1.

**Table 1.** Push-out test performed with 2.7 mm locking screws on the polyethylene surface.

Locking screw 2.7 mm – perpendicular to the surface		
Test no.	Torque (N cm)	Push-out force (N)
1.	150	897
2.	151	821
3.	151	811
4.	206	1138
5.	208	1089
6.	213	1317

**Figure 6.**
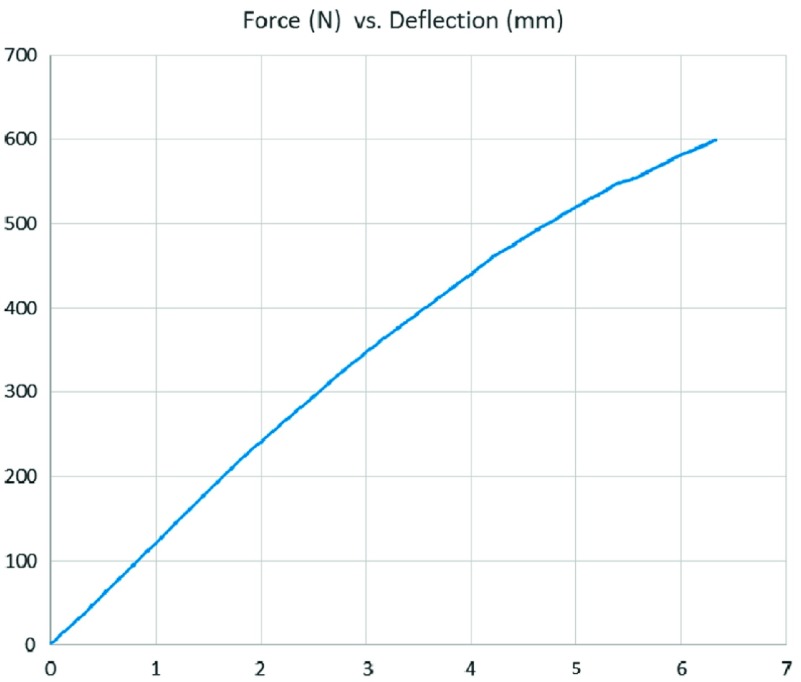
Force-deflection diagram of the shear force load (in N) plotted as a function of end deflection (in mm) of the Schanz screws inserted in the ONAS-DPS. The distance between the point force load and the fixation of the specimen was 60 mm.

Furthermore, the junction between Schanz screws and the ONAS-DPS was tested with applied shear load on a titanium specimen. The Zwick/Roell Z50 material testing machine was applying load cell of accuracy ±0.5 N and displacements were measured with accuracy of 0.02 mm. Figure 6 shows shear force load (in N) plotted as a function of end deflection (in mm) of the specimen. The distance between the point force load and the fixation of the specimen was 60 mm. Throughout the testing no visible failure was observed on the whole system, specifically on the contact between the bolt and the plate, which remained rigid by visual assessment for loads from 0 to 600 N.

## Discussion

Major complications of bone elongation include infection, poor control of elongation axis and mechanical failure of the automated IM nails. LON still seems to be the method of choice in most cases, especially in the femur. The newly developed ONAS-DPS enables LON with a major advantage of avoiding problematic screw insertion beside the IM nail. In particular, it is technically difficult to use the Schanz screws behind the IM nail in the trochanteric region in the case of an anterograde technique and in the metaphyseal region in the case of a retrograde technique. Besides, the screws should ideally be placed correctly (with the end result of adequate stable position in the bone) “within the first attempt”.

Fixation of Schanz screws to specially designed plates can be expected to lower the infection rate. In addition, a high degree of the system stability is achieved, which is crucial for elongation. ONAS-DPS enables a parallel position of the unilateral frame to be achieved owing to the varying assortment of the wedge subplates. Both anterograde and retrograde IM nail (Figure 1) can be used with ONAS-DPS. ONAS-DPS can also be used for elongation of the tibia. ONAS-DPS enables “aesthetic elongation”, since the distance between proximal and distal Schanz screws may be minimal. Both plates can be close together without any negative impact on the axis of elongation and in this way the unilateral frame may be very short.

ONAS-DPS retains all the advantages of LON over external fixation. Besides, it might also have advantages over automated IM nails: ONAS-DPS is patented and has the CE certificate. Preclinical studies are commencing and no suitable clinical data are available for publication at this moment.

## Conflict of interest

The implant is patented by: V. Antolič (2013) Modular side device with an intramedullary nail for guiding a bone during its lengthening. World Intellectual Property Organization. International Publication number: WO 2013/176632 A1.
